# Short-Term Hip Fracture Outcomes during the COVID-19 Pandemic

**DOI:** 10.1055/s-0041-1741511

**Published:** 2022-01-17

**Authors:** Samuel Walters, Hassan Raja, Rachel Ahmad, Konstantinos Tsitskaris

**Affiliations:** 1 Department of Trauma & Orthopaedic Surgery, Whipps Cross University Hospital, Barts Health NHS Trust, London, United Kingdom

**Keywords:** hip fracture, length of stay, mortality, 30-day mortality, COVID-19, SARS-CoV-2

## Abstract

**Introduction** Despite many significant changes as a result of the coronavirus disease 2019 (COVID-19) pandemic, and reductions in overall trauma workload, patients with fragility hip fractures continued to present to hospital. As we plan for ongoing service provision during future waves of the pandemic, valuable lessons can be learned from patients that have been treated surgically during the “first wave.”

**Methods** All patients admitted to our center (a busy District General Hospital in London, United Kingdom) with a hip fracture during a 13-week period representing the initial rise (“United Kingdom first wave”) in COVID-19 cases, from February 17 ^th^ to May 17 ^th^ , 2020 (study group) were compared with hip fracture patients from the equivalent 13-week period in February to May 2019 (control group). The primary outcome was 30-day mortality, and additional information was collected in terms of length of stay (LOS), SARS-CoV-2 antigen testing, and cause of death.

**Results** During the COVID-19 study period, 69 patients were admitted with a hip fracture, compared with 70 patients in the control group ( *p* = 0.949). There was no significant difference in 30-day mortality between the two groups (5.8 vs. 7.1%, *p* = 0.747). Mean LOS was shorter in the COVID-19 period compared with the control group (11.6 vs. 19.6 days, *p* <0.001, effect size 0.572).

Forty-six patients (66.7%) had a SARS-CoV-2 antigen swab test, as testing was not available in the early period, and 10 patients (14.5%) tested positive. None of the patients, who presented before the antigen testing was available, had clinical suspicion of COVID-19 retrospectively. Two “COVID-19 positive” patients (20%) died within 30 days of admission.

**Conclusion** We report reassuring short-term results demonstrating no statistically significant difference in the 30-day mortality rate of hip fracture patients admitted during the United Kingdom's first wave of the COVID-19 pandemic compared to the equivalent period in the previous year. Hip fracture incidence remained stable, and LOS was reduced, likely due to recent departmental changes as well as a drive to discharge patients quickly during the pandemic. We agree with existing reports that elderly hip fracture patients with COVID-19 have a higher risk of perioperative mortality, however, our results suggest that overall mortality for the whole hip fracture population was similar to the previous year, in which deaths were more commonly attributed to respiratory infections associated with other pathogens. Further work may be needed to evaluate the outcomes during subsequent waves of the pandemic as mutations in the virus and conditions may affect outcomes.

The first confirmed case of the novel coronavirus in the United Kingdom was on January 30 ^th^ , 2020, however, the effects were predominantly felt from March 2020 onward. London was widely regarded as the epicenter of the initial outbreak (“first wave”) in the United Kingdom, and was the region with the highest total number of confirmed cases during this period, with the peak hospital admissions and deaths occurring in April 2020. Since then, further waves have affected a wider geographical distribution throughout the United Kingdom. 1

Hip fractures are a cornerstone of trauma workload in Trauma and Orthopaedic Surgery; they are markers of frailty and usually affect patients with multiple medical problems. Early during the pandemic, it was recognized that the prevalence of hip fractures was unlikely to decrease, and it was possible that it could even increase on account of their usual mechanism (fall from standing height at home) and decreased social support. Individual units developed tools and pathways, in order to mitigate potential shortfalls in theatre capacity and availability of resources. A shift from “sickest first” to “fittest first” was considered, as along with the option of non-operative treatment for the frailest patients, particularly if medically compromised as a result of COVID-19. 2

In response to the rapidly changing situation and unprecedented requirements on the health care service, elective and planned surgical work was stopped, but the provision of an emergency service for patients with traumatic injuries continued, with significant procedural changes in relation to personal protective equipment and logistical considerations in theatre.

There was a noticeable decrease in other traumatic injuries during this period, due to lifestyle changes during the lockdown, 3 however, the delivery of surgical care for patients with hip fractures was one of the very few areas of practice that remained relatively unaffected in terms of the volume of cases and surgical management. 4 5 As a result, the outcomes in this group of patients can be a useful resource in better understanding the risks involved in delivering surgical treatments during the COVID-19 pandemic. Hip fracture patients tend to be frail and often poorly optimized when compared with candidates for elective arthroplasty. In spite of this, these patients undergo major orthopaedic surgical interventions, and they are currently the most consistent cohort of patients to extrapolate outcomes from, and may provide useful information as we plan to return to normal activities internationally. 6

We aimed to evaluate the 30-day mortality of patients with hip fractures treated in a busy District General Hospital in London, during the initial peak (“first wave”) of the COVID-19 pandemic. Additionally, this study aimed to review outcomes in relation to SARS-CoV-2 antigen swab tests and clinical suspicion of COVID-19, as well as length of stay (LOS).

## Methods

### Study Design

This was a retrospective observational study at a district general hospital in London, United Kingdom. This was a service evaluation exercise and did not require ethical approval. We defined the study period as February 17 ^th^ to May 17 ^th^ , 2020 (study group). This 13-week period was chosen as it includes the significant initial rise of coronavirus cases from late February onward, the peak seen in early April and the gradual decrease in cases later on. The control group was defined as the same 13-week period in the previous year: February 18 ^th^ to May 19 ^th^ , 2019.

### Study Population

All adult patients managed by our service with a proximal femoral fracture were included. This included intracapsular, trochanteric, and subtrochanteric fractures. Children, femoral shaft fractures, distal femoral fractures, and periprosthetic fractures were excluded.

### Data Collection

Patients were identified for the study period using our prospectively maintained trauma database, and electronic notes were reviewed to determine outcomes including LOS, mortality, and SARS-CoV-2 antigen swab testing. The control group was identified from our database of hip fracture patients from 2019, compiled for the National Hip Fracture Database (NHFD). We evaluated outcomes for the whole year of 2019, as well as for a period spanning from February 18th to May 19 ^th^ , 2019 (control group).

### Statistical Analysis

Continuous variables (age, comorbidities, and LOS) were presented as mean, standard deviation, median and range, and checked for normality using a Shapiro-Wilk test. Results were compared using an independent samples *t* -test (Student test for parametric data, Mann-Whitney U test for non-parametric data) and effect size was calculated with rank-biserial correlation in the case of non-parametric data. Categorical variables (30-day mortality, gender) were presented as frequencies and percentages, and were compared using a Chi-square test and Phi coefficient to estimate effect size. Incidence was compared using a Chi-square test. JASP, Version 0.13.1 (University of Amsterdam, Netherlands) 7 was used for statistical analysis.

## Results

### Incidence, Demographics, 30-Day Mortality, and LOS

#### Study Group (2020 Data)

Between February 17th and May 17 ^th^ , 2020, our service admitted 69 patients with hip fractures. Forty-eight of these (69.6%) were female, and the mean age was 83.1 (SD = 11.1, median = 84.5, range = 40–99). The mean number of comorbidities was 3.0 (SD = 1.6, median = 3, range = 0–8).

The 30-day mortality rate was 5.8% (four deaths), with a mean time from admission to death of 17.7 days (SD = 6.4 days, median = 19 days, range 9.2–23.4 days). The remaining 65 patients had a mean LOS of 11.6 days (SD = 8 days, median = 10 days, range = 1.9–61 days).

#### Control Group (2019 Data)

In the whole of 2019, our service treated 326 patients with a hip fracture, and the 30-day mortality rate was 6.13% (20 deaths). In the control group (February 18th to May 19 ^th^ , 2019) there were 70 cases, of which 47 were female (67.1%); the mean age was 83.6 (SD = 8.7, median = 84.8, range = 63–98), and the mean number of comorbidities was 2.9 (SD = 1.3, median = 3, range = 1–6). There were no statistically significant differences between number of cases (69 vs. 70, *p* = 0.949), gender (69.6 vs. 67.1% female, *p* = 0.759), mean age (83.1 vs. 83.6, *p* = 0.838), or mean number of comorbidities (3.0 vs. 2.9, *p* = 0.978).

The 30-day mortality rate was 7.1% (five deaths), with a mean time from admission to death of 11.2 days (SD = 10.6 days, median = 6.9 days, range = 3.6–29.3 days). The remaining 65 patients had a mean LOS of 19.6 days (SD = 11.3 days, median = 17.4 days, range = 7.7–67.5 days). For both groups, the results for number of comorbidities, age, and LOS did not follow a normal distribution (Shapiro-Wilk *p* <0.05), so the Mann-Whitney U test was used in all cases.

There was no statistically significant difference between the 30-day mortality rates in the study and control groups (5.8 vs. 7.1%, *p* = 0.747, Phi coefficient 0.027). The mean LOS was significantly shorter in the study group compared with the control group (11.6 vs. 19.6 days, *p* <0.001, effect size 0.572—medium to large effect). The mean LOS for the whole of 2019 was 18.2 days, which was also significantly longer than in the study group (11.6 vs. 18.2, *p* <0.001, effect size 0.389), and similar to the control group (19.6 vs. 18.2, *p* = 0.086, effect size 0.137). The results are summarized in Table 1 .

**Table 1:** Hip fracture incidence, age, gender, number of comorbidities, 30-day mortality and mean length of stay during the COVID-19 period in 2020 compared with the equivalent period in 2019

	Study group (COVID-19) *February–May 2020*	Control group *February–May 2019*	
Number of patients admitted with hip fracture	**69**	**70**	*p* = 0.949
Age (mean ± SD, [range])	**83.1** ± 11.1, [40–99]	**83.6** ± 8.7, [63–98]	*p* = 0.838
Female	**69.6%** (48)	**67.1%** (47)	*p* = 0.759
Number of comorbidities (mean ± SD, [range])	**3.0** ± 1.6, [0–8]	**2.9** ± 1.3, [1–6]	*p* = 0.978
30-day mortality	**5.8%** (4)	**7.1%** (5)	*p* = 0.747
Mean length of stay (days) (mean ± SD, [range])	**11.6** ± 8, [1.9–61]	**19.6** ± 11.3, [7.7–67.5]	*p* <0.001

#### SARS-CoV-2 Testing

During the study period, 46 patients (66.7%) were tested for SARS-CoV-2 using throat and nose swab assay with RT-PCR for SARS-CoV-2, with the first test performed on March 22 ^nd^ , 2020. Out of the remaining 23 patients that never had an antigen swab test, 18 (78.2%) were admitted in the first 4 weeks of the study period (February 17th–March 15 ^th^ , 2020). The testing capacity and relevant protocols evolved during this period of the pandemic. From mid-March to mid-April, patients were tested only if symptomatic, whereas from mid-April onward, there was universal testing on admission and periodic retesting during the hospital stay. Patients admitted between March 26 ^th^ , 2020 and the end of the study period, had a mean of 2.3 tests during their hospital stay.

Ten patients (14.5%) tested positive for SARS-CoV-2. In terms of isolation and supportive measures, they were treated in accordance with the trust policy at the time. None of the patients, who presented before the antigen testing was available, had clinical suspicion of COVID-19 retrospectively. Patients who tested negative, but had symptoms suggestive of COVID-19, were isolated and repeatedly tested every few days during the admission. If these repeated tests were negative, they were considered not to have had COVID-19. Of the 10 “COVID-19 positive” patients, two (20%) died within 30 days of admission (days 16 and 23), both from respiratory failure. Three more “COVID-19 positive” patients died beyond the 30-day threshold, two from respiratory failure on days 38 and 49, and the third following a cerebrovascular event on day 92. All of these patients had significant comorbidities, advanced age, or a combination of both. Further information is presented in Table 2 .

**Table 2:** Demographic information, COVID-19 status, and cause of death for mortalities during the study period

No.	Age	Gender	Comorbidities	COVID-19 status	Days from admission to death	Cause of death
***Mortalities within 30 d***						
1	93	F	AF, Severe mitral regurgitation, HTN, previous contralateral hip fracture (Hemiarthroplasty)	Negative (not tested) *Admitted 20th February*	22	Gradual decline postoperatively from congestive cardiac failure and hospital-acquired pneumonia. Switch to palliative approach, transferred to palliative unit on day 20
2	88	F	Dementia, HTN, colitis, macular degeneration	Negative (not tested) * Admitted 27 ^th^ February *	9	Postoperative chest infection, with continued deterioration despite antibiotics. Hypoactive delirium and presumed aspiration.
3	88	M	AF, IHD (previous stents), HTN, COPD, T2DM, chronic kidney disease, prostate cancer, colitis	Positive (1 ^st^ swab)— * 26 ^th^ March *	23	Postoperative pneumonia, failure to respond to IV antibiotics, COVID-19 positive on day 16 post-operatively. Gradual decline despite ward-based supportive treatment (not a candidate for positive pressure treatment). Switch to palliative approach, died from COVID-19
4	75	M	Dementia, COPD	Positive (1 ^st^ swab)— * 22 ^nd^ March *	16	Initial good recovery. On day 9 postoperatively became pyrexial and tachypneic. SARS-CoV-2 swab taken (positive) and protocols followed. Gradual decline despite supportive treatment, died from COVID-19
*COVID-19 positive patients with mortality after 30 d*						
5	83	M	Dementia, IHD, HTN, hypercholesterolemia	Positive (1 ^st^ and 2 ^nd^ swabs)— * 12 ^th^ April, 30 ^th^ April * 3 ^rd^ swab negative— * 3 ^rd^ May *	38	Discharged to inpatient rehabilitation on day 6. Readmitted under the medical team on day 26 with chest pain. Desaturation and COVID-19 positive. Gradual decline despite supportive treatment and switch to palliative approach.
6	99	F	Dementia, lacunar infarct, HTN, gastroesophageal reflux disease, hypercholesterolemia	Positive (2 ^nd^ , 3 ^rd^ and 4 ^th^ swabs)— * 8 ^th^ April, 10 ^th^ April, 21 ^st^ April * During re-admission under medical team.	49	Discharged to Residential Home on day 10. Readmitted on day 23 under medical team with worsened confusion—desaturation requiring oxygen, and hyponatremia. Gradual deterioration despite supportive treatment. Died from COVID-19.
7	89	F	Two previous cerebrovascular events in previous 3 years. Fall caused by further cerebrovascular event (bilateral MCA infarcts).	Positive (3 ^rd^ swab)— * 20 ^th^ April * 1 ^st^ , 2 ^nd^ , 4 ^th^ , and 5 ^th^ swabs negative. Was considered to have recovered from COVID-19 prior to death.	92	Poor functional recovery post-operatively, hospital acquired pneumonia + COVID-19 positive. Transferred back to stroke ward. Recovered from chest infection, but gradual decline and switch to palliative approach. Died from cerebrovascular event.

Abbreviations: AF, atrial fibrillation; COPD, chronic obstructive pulmonary disease; HTN, hypertension; IHD, ischemic heart disease; T2DM, type 2 diabetes mellitus.

## Discussion

We present a reassuring overview of the outcomes of hip fracture patients admitted to our unit, during the first wave of the COVID-19 pandemic in the United Kingdom. There was no statistically significant difference in the 30-day mortality rate of patients admitted during this period compared to the equivalent period in the previous year (5.8 vs. 7.1%, *p* = 0.747). During the two 13-week study periods in 2020 and 2019, there were near identical numbers of admissions (69 and 70, respectively, *p* = 0.949), suggesting that the pandemic did not influence the incidence of hip fractures.

The NHFD was established in 2007 and gathers national data for patients with hip fractures in the United Kingdom. The annual reports include outcomes of key performance indicators and mortality rates. The national 30-day mortality for patients presenting with hip fractures is 6.1% (2019 NHFD Report based on 2018 data). 8 There is a well-recognized seasonal variation of mortality following hip fractures, with higher rates expected during the winter months. 8 We believe that it was important to mitigate this seasonal bias, hence, we elected to compare the outcomes of the study group to the corresponding period in 2019. The 30-day mortality in the study group (5.8%) was in keeping with the expected seasonal values, as well as being in line with the 30-day mortality in our unit for the whole of the previous year.

Sobti et al reported similar findings for hip fracture patients in Surrey, United Kingdom, during the period from March to May 2020, with 8.46% mortality, and no statistically significant difference in mortality compared to a similar period in 2019, however, this group only contained three “COVID-19 positive” patients, suggesting a much lower prevalence of the virus in this region at the time. 9 Similarly, Malik-Tabassum et al reported no statistically significant differences in mortality for hip fracture patients in East Sussex, United Kingdom, during this period, but only had one “COVID-19 positive” patient in the cohort. 10

When evaluating the “COVID-19 positive” patients in isolation, we found a 30-day mortality of 20%, increasing to 50% when followed up over a longer period (up to 92 days). Cheung and Forsh reported on a series of 10 COVID-19 positive patients with hip fractures, of whom two had respiratory symptoms and eight were asymptomatic. All the patients underwent surgery within 2 days. There was one death (10%), secondary to presumed venous thromboembolism and respiratory failure, affecting one of the two symptomatic patients. Although this is a very small cohort, the authors report that asymptomatic COVID-19 positive patients, can undergo surgical treatment without a dramatically increased risk of mortality. 11

Chui et al reported outcomes following the implementation of a split-site protocol in a large district general hospital in London. During the period from March 31 ^st^ to April 30 ^th^ , 2020, they treated 47 patients with hip fractures, 12 of whom were considered COVID-19 positive (six with positive tests and six with negative tests but with high clinical suspicion). The remaining 35 “non-COVID” patients were transferred to a “COVID-free” site in the independent sector. The early mortality rate for the “non-COVID” group was 5.7%, compared to 25% for the COVID-19 positive group (three deaths). The latter group was recognized as being inherently at a higher risk based on their medical background. There was no statistically significant difference in the mortality between the two groups ( *p* = 0.0971) and the overall mortality reported was 10.6% at a mean follow-up of 24.7 days. 12

Our findings do not disagree with the multiple reports showing an association between COVID-19 infection and mortality in hip fracture patients, 13 14 15 16 17 18 19 as well as reports of an association between frailty and mortality in COVID-19 positive patients. 20 In our series, the group of patients who succumbed to COVID-19 pneumonia was characterized by a background of significant comorbidities, very advanced age, or a combination of both. These are typically high-risk patients who, in any given year, have a high risk of succumbing to a respiratory infection perioperatively. In previous years the most prevalent pathogen was not well defined, but in 2020 the most prevalent pathogen was SARS-CoV-2. Irrespective of the causative microorganism, the overall early mortality rate remained consistent in our department.

LOS was significantly shorter for the COVID-19 group, both when compared to the control group and the whole of the 2019 cohort. During the months leading to the pandemic, we recorded significant improvements to the mean LOS in our unit. This was a result of the implementation of new pathways whilst our service was going through a period of transformation, aiming to become a hip fracture hub for East London. The trend for an improved mean LOS possibly contributed to the short LOS recorded during the pandemic, but it is unlikely to be the sole reason for it; a national drive to discharge patients from acute beds very likely contributed too.

This study has limitations. Firstly, it is a retrospective look of short-term outcomes of hip fracture patients receiving surgical treatment during the first wave of the pandemic. Nevertheless, 30-day mortality is one of the most important indicators of perioperative performance and was, hence, selected as a primary outcome in this study. Secondly, hip fracture patients with their inherent high risk, are not representative of all other patient groups. However, in the context of the coronavirus pandemic, they were one of the very few groups of patients whose number and treatment were not dramatically altered, and can be a valuable source of information. Thirdly, the numbers in this study are fairly small on account of short study periods (two 13-week periods), but this was a necessary compromise to make in order to succinctly study the period of interest without a long period of normality either side.

At the time of writing, there are ongoing pressures in relation to COVID-19 internationally, with considerable variability between countries, which undoubtedly continues to impact on the care and outcomes for all patients including those with hip fractures. Further work is needed to characterize the outcomes for patients in the later stages of the pandemic, as well as any differences which may be present as a result of mutations in the virus and different environmental factors such as colder weather over the winter months.

## Conclusion

We report reassuring short-term results demonstrating no significant difference in the 30-day mortality rate of hip fracture patients admitted during the United Kingdom first wave of the COVID-19 pandemic in 2020, and no increase in LOS. Our findings agree with existing reports that elderly hip fracture patients with COVID-19 indeed have a high risk of perioperative mortality, however, our data suggests that overall mortality was similar in previous years, in which deaths were more commonly attributed to respiratory infections associated with other pathogens.
